# Proteomics Data Imputation With a Deep Model That Learns From Many Datasets

**DOI:** 10.1016/j.mcpro.2025.101461

**Published:** 2025-11-18

**Authors:** Lincoln Harris, William S. Noble

**Affiliations:** 1 Department of Genome Sciences, University of Washington, Seattle, Washington, USA; 2 Paul G. Allen School of Computer Science and Engineering, University of Washington, Seattle, Washington, USA

**Keywords:** proteomics, mass spectrometry, imputation, machine learning, deep learning

## Abstract

Missing values are a major challenge in the analysis of mass spectrometry proteomics data. Missing values hinder reproducibility, decrease statistical power for identifying differentially abundant proteins, and make it challenging to analyze low-abundance proteins. We present Lupine, a deep learning–based method for imputing, or estimating, missing values in quantitative proteomics data. Lupine is, to our knowledge, the first imputation method that is designed to learn jointly from many datasets, and we provide evidence that this approach leads to more accurate predictions. We validated Lupine by applying it to tandem mass tag data from *>*1000 cancer patient samples spanning 10 cancer types from the Clinical Proteomics Tumor Atlas Consortium. Lupine outperforms the state of the art for proteomics imputation, uniquely identifies differentially abundant proteins and Gene Ontology terms, and learns a meaningful representation of proteins and patient samples. Lupine is implemented as an open-source Python package.

## Highlights

• Lupine is a deep learning model that learns jointly from many mass spectrometry datasets and imputes missing values.
• Lupine outperforms state-of-the-art imputation methods in terms of quantitative accuracy.
• Lupine recovers expected differentially abundant proteins in simulated data and identifies more differentially abundant proteins than competing methods in cancer proteomics data.
• Lupine learns a rich and biologically meaningful embedding of cancer proteomics data from Clinical Proteomics Tumor Atlas Consortium.
• Lupine performs well on tandem mass tag, data-independent acquisition, and data-dependent acquisition label-free quantification proteomics data.

## Graphical Abstract

**Figure:**
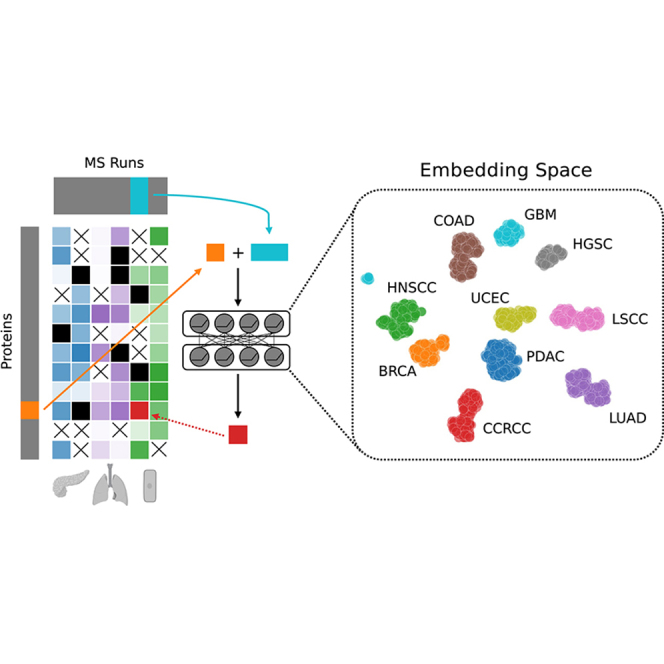

## In Brief

Analysis of mass spectrometry proteomics data is limited by missing values, which can contribute to lower statistical power, inability to quantify low-abundance proteins, and loss of potentially important differential proteins. We developed a state-of-the-art deep learning–based model for imputing, or estimating, missing values in quantitative proteomics. Better tools for handling missing values will allow researchers to extract more information from large-scale quantitative proteomics experiments, leading to more discoveries in fields, such as cancer biology, neurodegeneration, and aging.

In spite of significant advances in instrument technology and sample preparation, missingness remains a challenge in quantitative mass spectrometry (MS) proteomics (1, 2). “Missingness” refers to proteins that are present in the analyte, but for various technical reasons, including coelution, electrospray competition, and inability to confidently assign peptide spectrum matches, these proteins do not have quantitative values (1, 2). Missingness hinders reproducibility and reduces statistical power, making it difficult to compare across runs or experimental conditions. In addition, missingness can make it challenging to glean information from low-abundance proteins, which are important for the etiology and progression of a number of diseases (3, 4, 5).

Empirically, missing values are not distributed entirely randomly but instead are typically correlated with the intensity of the protein (6). In general, missing values may be either missing completely at random (MCAR) or missing not at random (MNAR) (7). In the case of MCAR, there is no relationship between the underlying variables and the likelihood of an observation being missing, whereas for MNAR, there is. In MS proteomics, missing values tend to be MNAR because the likelihood that a protein is missing depends on its intensity (6). Low-intensity proteins are more likely to be missing, although medium- or high-intensity proteins are occasionally missing as well (6). The reasons for this vary by acquisition strategy—in tandem mass tag (TMT), high-intensity proteins may be missing because of stochastic sampling between acquisitions, whereas in data-independent acquisition (DIA), all precursor ions within an isolation window are fragmented, leading to substantially less missingness among high-intensity proteins.

Here, we focus primarily on missing values in TMT proteomics. Missingness is especially pronounced for large-scale, multibatch TMT experiments, in which the number of missing values scales logarithmically with the number of TMT batches or “plexes” (8). Nevertheless, TMT offers unparalleled quantitative accuracy and has recently been used for large-scale studies of cancer and neurodegenerative disease (9, 10, 11, 12, 13). For example, TMT was used by the Clinical Proteomics Tumor Atlas Consortium (CPTAC) project (10, 13) to analyze more than 1000 clinical patient samples from 10 types of cancer.

Imputation is an analytical solution to missingness. “Imputation” refers to the use of statistical or machine learning procedures to estimate missing values based on the observed values alone. Imputation is routinely used to handle missingness in data from microarrays (14), single-cell transcriptomics (15, 16), epidemiology (17), and astronomy (18, 19). There are many methods for proteomics imputation; however, each of them has significant limitations. For example, the majority of these methods have been borrowed from microarray analysis and were not specifically developed for MS. The most commonly used method is Gaussian random sampling, in which imputed values are drawn from a Gaussian distribution centered on the lowest observed quantification (20). This method is used by the popular Perseus tool (21) for MS data analysis. Despite its popularity, Gaussian random sampling works poorly (20).

DreamAI is a state-of-the-art method for MS proteomics imputation (22). DreamAI is an ensemble of the six winning methods from the National Cancer Institute–CPTAC DREAM challenge (https://www.synapse.org/Synapse:syn8228304/wiki/413428), in which more than 20 teams competed to develop imputation methods for CPTAC TMT and isobaric tag for absolute and relative quantification data. DreamAI ensembles several high-performing individual methods, including MissForest (23) and *k*-nearest neighbors (kNN) (14). The DreamAI ensembling strategy outperforms any of its six individual methods (22).

Deep learning (DL) has revolutionized our ability to analyze biological data. Most famously, DL is being used to predict protein structures and to discover novel drug targets (24). Within the field of proteomics, DL has been used for spectral library generation (25), retention time prediction (26) and peptide *de novo* sequencing (27). One general feature of most DL methods is that they benefit from training with as much data as possible. For example, DL-based *de novo* sequencing methods have been trained on 30 million peptide-spectrum matches from MassIVE-KB (27).

In spite of its impressive performance in other domains, DL has not yet gained widespread adoption for proteomics imputation. To our knowledge, there exists only one DL-based proteomics imputation method, called PIMMS, developed for label-free quantification (LFQ) (28). Perhaps one explanation for the lack of adoption of DL is that existing strategies consider only a single dataset at a time and therefore do not benefit from very large training sets derived from multiple MS experiments. In this context, underfitting is always a concern, especially when attempting to fit large DL models with many parameters.

Here, we present Lupine, a DL-based proteomics imputation method, which learns patterns of missingness from multiple datasets simultaneously. We trained Lupine on a joint quantifications matrix that consisted of proteins and MS samples from 10 different TMT datasets. Lupine learns low-rank protein and sample embeddings, which are fed into a deep neural network (DNN) to generate predictions. Lupine incorporates an MNAR assumption into its training procedure, explicitly assuming that most missingness is left censored. Our experiments demonstrate that Lupine’s performance improves when trained on 10 datasets as opposed to one. Lupine outperforms the current state of the art in terms of quantification accuracy and identifies more differentially abundant (DA) proteins than competing methods. In addition, Lupine learns a meaningful latent representation of proteins and MS samples. While we focus primarily on TMT, we also show that Lupine performs well for other proteomics modalities, including DIA and data-dependent acquisition LFQ. We stress that Lupine is not limited to cancer proteomics and may be applied to peptide or protein quantitative data from any acquisition strategy or sample type.

## Experimental Procedures

### Joint Quantifications Matrix Construction

We obtained TMT proteomics data—collected by CPTAC—through the Proteomics Data Commons web portal (https://pdc.cancer.gov/pdc/cptac-pancancer, file name: Proteome UMich GENCODE34v1.zip). Data were collected at five centers: Pacific Northwest National Laboratories, Vanderbilt University, Johns Hopkins University, and the Broad Institute. Samples were run on Thermo Fisher Orbitrap instruments. Datasets were processed with the same analytical pipeline, the full details of which are provided in the STAR Methods of Li *et al*. (10). Briefly, this pipeline consisted of peptide search with MSFragger (29) against a GENCODEv34 database and postprocessing with Philosopher (30) and TMT-Integrator (29). TMT-10 or TMT-11 plexes were normalized to a common reference channel and then summarized at the protein level.

We constructed a joint quantifications matrix from CPTAC datasets. Rows in the joint quantifications matrix were proteins, and columns were demultiplexed TMT samples. When adding new MS samples (*i.e.*, columns) to the matrix, if a protein was not quantified in the given sample, then the matrix entry was assigned a value of NaN (not a number) to indicate a missing value. We removed proteins quantified in fewer than 18 samples. Sample IDs containing the following key words were excluded from analysis: “RefInt,” “QC,” “pool,” “pooled,” “reference,” “NCI,” “NX,” and “ref.” After filtering, this dataset consisted of 18,162 proteins and 1755 samples. The overall missingness fraction was 48.7%.

Protein quantification matrices for the ProCan (31) and mechanism of action (MoA) (32) datasets were obtained from PRIDE (PXD030304 and PXD018569, respectively). For ProCan, library search was performed with DIA-NN (33) and quantification with MaxLFQ (34). For MoA, MaxQuant (35) was used for library search. Lupine was fit to the ProCan and MoA datasets individually; that is, the joint training procedure was not used.

Details of the TMT, DIA and LFQ datasets used for model training are provided in Table 1.

**Table 1:** Description of the datasets used in this study

Dataset	Type	*N* proteins	*N* samples	Percent missing (%)	Citation
BRCA	TMT	12,825	153	21.4	Li *et al.* (2023) (6)
CCRCC	TMT	11,821	194	20.7	Li *et al.* (2023) (6)
COAD	TMT	9433	197	24.6	Li *et al.* (2023) (6)
GBM	TMT	12,875	110	15.9	Li *et al.* (2023) (6)
HGSC	TMT	10,967	103	21.5	Li *et al.* (2023) (6)
HNSCC	TMT	12,158	188	19.3	Li *et al.* (2023) (6)
LSCC	TMT	13,625	215	16.8	Li *et al.* (2023) (6)
LUAD	TMT	13,206	221	18.4	Li *et al.* (2023) (6)
PDAC	TMT	11,968	239	23.6	Li *et al.* (2023) (6)
UCEC	TMT	12,505	135	18.9	Li *et al.* (2023) (6)
ProCan	DIA	12,576	1000	62.5	Gocalves *et al.* (2022)
MoA	LFQ	6738	180	22.2	Ruprecht *et al.* (2020) (32)

BRCA, breast cancer; CCRCC, clear cell renal cell carcinoma; COAD, colon adenocarcinoma; GBM, glioblastoma; HGSC, high-grade serous carcinoma; HNSCC, head and neck squamous cell carcinoma; LSCC, lung squamous cell carcinoma; LUAD, lung adenocarcinoma; PDAC, pancreatic ductal adenocarcinoma; UCEC, uterine corpus endometrial carcinoma.

The overall percentage of missing entries across each protein quantifications matrix is reported.

### Dataset Partitioning and Batch Selection

We used an MNAR data partitioning procedure to simulate the type of missingness most common to MS proteomics (1, 2, 6). The goal was to withhold a test set that was left-shifted (*e.g.*, with a lower mean) than the training set, to mimic the setting in which there is more missingness among low-abundance proteins. Given our joint quantifications matrix *X* with rows *i* and columns *j*, we constructed a “thresholds” matrix *T*_*i×j*_. *T* was populated with values sampled from a Gaussian distribution centered about the 25th percentile of *X* and with standard deviation (*σ*) 1.1 × *X*_*σ*_. For each *X*_*ij*_, if the corresponding *T*_*i×j*_ < *X*_*ij*_, then *X*_*ij*_ was assigned to the training set. Otherwise, a Bernoulli trial with success probability 0.61 was conducted. If the trial was successful, then *X*_*ij*_ was assigned to the test set; otherwise, *X*_*ij*_ was assigned to the training set. The Bernoulli success probability and Gaussian distribution mean and standard deviation were tuned such that 20% of present matrix entries were assigned to the test set and the remaining 80% were assigned to the training set. The end result was a test set with a lower mean than the training set.

We implemented a “biased” batch selection procedure during model training. To achieve this, we conducted multiple rounds of the Bernoulli trial–based selection procedure described above, in which the training set was sampled with replacement to create a series of training batches. Low-intensity proteins were preferentially selected for training batches (supplemental Fig. S1). The resulting distribution of training batches is left skewed relative to the whole training set. The test set remained hidden during this procedure.

### Model Implementation

**Fig. 1:**
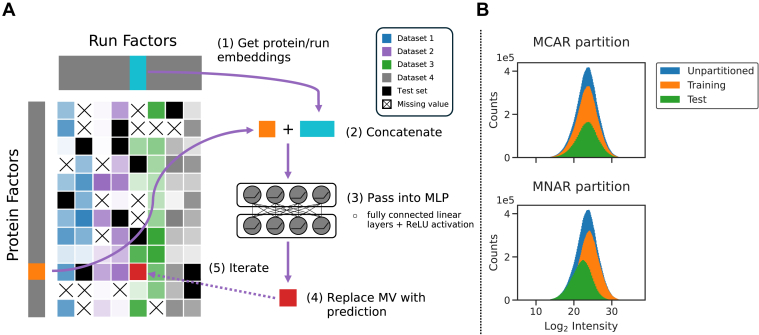
**Lupine schematic and data partitioning procedure.***A,* model schematic. Many datasets were combined into a single joint quantifications matrix to which Lupine was fit. Here, the intensity of each color in the joint quantifications matrix corresponds to protein intensity. *B,* illustration of standard MCAR *versus* Lupine’s MNAR data partitioning schemes. Lupine was trained entirely in the MNAR setting. MCAR, missing completely at random; MLP, multilayer perceptron; MNAR, missing not at random.

Lupine uses a DNN to learn a low-dimensional representation of proteins and MS samples (Fig. 1*A*). The protein and sample factor matrices, referred to as *W* and *H*, respectively, were randomly initialized linear-embedding layers. The shapes were as follows: *W*: (*n*, *p*) given *n* proteins and *p* protein factors and *H*: (*q*, *m*) given *q* sample factors and *m* samples. *W* and *H* contained no missing values. Selection of model hyperparameters *p* and *q* is described in the next section.

The Lupine training procedure was as follows. For each training batch, for each *Xtrain*_*i,j*_, the corresponding protein (*W*_*i*_) and sample (*H*_*j*_) factors were concatenated and fed into a fully connected multilayer perceptron (*i.e.*, the DNN). This DNN consisted of a variable number of hidden layers and nodes per layer, with leaky ReLU activations (negative slope 0.1) after each layer. The DNN outputs a predicted value *Xpred*_*i,j*_. The mean squared error (MSE) between *Xtrain*_*i,j*_ and *Xpred*_*i,j*_ was calculated. This procedure was repeated for every *Xtrain*_*i,j*_ in the train batch, and then model parameters *W*, *H*, and the DNN weights and biases were updated with backpropagation. The Adam optimizer was used, with a learning rate of 0.001. The training batch size was 128.

A validation set was used to determine model convergence. This validation set consisted of 10% of matrix entries, MCAR selected from the training set. Training proceeded until one of two stopping criteria was triggered, as evaluated on the validation set: (1) The ratio of the difference between the best loss and the current loss (numerator) to the best loss (denominator) was calculated at the end of each epoch. If this ratio was less than a tolerance parameter (0.001) for 10 successive epochs, then training stopped. (2) A Wilcoxon rank sum test was conducted between two “windows” of validation set MSEs, window 1 being the previous five training epochs and window 2 being training epochs *n −* 10 to *n −* 15, where *n* is the current epoch. If the one-sided Wilcoxon *p* value comparing window 2 to window 1 was *<*0.05, then training stopped, indicating that validation error had stopped decreasing and had started to increase.

Lupine was implemented in PyTorch, version 1.10.2 and was trained on a combination of NVIDIA GeForce GTX 1080, GeForce RTX 2080, and GeForce RTX 2080 Ti graphic processing units.

### Benchmarking

The CPTAC joint quantifications matrix was partitioned into 80% train and 20% test with an MNAR procedure, as described in the “Dataset Partitioning and Batch Selection” section. Proteins with fewer than 18 present values in the training set were removed from the training and test matrices. This cutoff reflects our desire to include as many proteins as possible while recognizing that proteins with too few present values will result in low-quality imputations. This partitioning procedure was repeated for the ProCan and MoA datasets.

For the CPTAC data, the training set was then divided into 10 subsets, each containing the MS samples for a single CPTAC cohort. For each cohort subset, proteins with fewer than three present values were removed from the matrix. Baseline methods, including DreamAI, MissForest, kNN, and Gaussian random sampling imputation, were fit to each cohort subset. DreamAI was accessed *via* GitHub (https://github.com/WangLab-MSSM/DreamAI) and was run in R software. The MissForest CRAN package was used. The scikit-learn implementation of KNNImputer (version 1.5.2) was used, where features were proteins and observations were MS runs. Gaussian random sampling was implemented with custom Python code replicating the Perseus procedure described here: https://cox-labs.github.io/coxdocs/replacemissingfromgaussian.html.

Lupine is an ensemble of individual models, each trained with different values for the following hyperparameters: number of protein factors *p*, number of sample factors *q*, number of hidden layers, and number of nodes per hidden layer. The search space for each hyperparameter was as follows: *p* and *q*: [64, 128*,* 256, 512, and 1024], hidden layers: [1, 2, and 4], and nodes per hidden layer: [512, 1024, and 2048]. Of the 225 possible combinations of hyperparameters, a random selection of 42 was chosen. *n* = 42 independent Lupine models were then fitted to the full training matrix. Each model used a different random seed and partitioned a different 10% of the training set *X*_*ij*_s into its validation set. The predictions from these 42 models were then averaged to generate the final Lupine-reconstructed matrix.

To enable one-to-one performance comparisons, for each cohort, the Lupine-reconstructed matrix was subset to include only the proteins contained by the baseline method’s training matrix for that cohort. In this way, we evaluated predictions on the same set of proteins and MS samples for every model. We calculated the MSE between model predictions and the observed test set values for each cohort.

For the ProCan and MoA benchmarking experiments, MissForest was limited to four training iterations for the sake of computational tractability. In addition, benchmarking was limited to the first 1000 runs of the ProCan dataset (6980 total runs).

### Differential Abundance

For each CPTAC cohort, we identified DA proteins between tumor and nontumor samples both with and without imputation. Lupine, DreamAI, and Gaussian random sampling imputation were performed as described in the previous section. A comparison to *no imputation* was also included. Metadata were obtained from the CPTAC data portal: https://pdc.cancer.gov/pdc/. For each cohort, proteins with *>*50% missingness prior to imputation were excluded from DA analysis, which is a common practice prior to DA analysis in quantitative proteomics studies (36, 37).

For each imputed protein, paired *t* tests were conducted between protein quantifications from tumor and nontumor samples. For the *no imputation* comparison, missing values were simply ignored when conducting *t* tests. *P* Values were adjusted with the Benjamini–Hochberg procedure (38). Proteins with Benjamini–Hochberg–adjusted *p* values *<*0.01 and log_2_ fold changes *>*0.5 were considered DA.

To identify enriched Gene Ontology (GO) terms, we used the PANTHER over-representation test (https://pantherdb.org/tools/compareToRefList.jsp). PANTHER19.0 was used. For each cohort, the set of upregulated DA proteins was compared with a background list consisting of all 18,162 proteins in the joint quantifications matrix. The test type was Fisher’s exact, and the annotation dataset was “GO biological process complete.” Gene sets with *q* values below 0.05 were considered significant.

### Simulation

We simulated protein quantification data to assess Lupine’s ability to identify known DA proteins. Two empty arrays *A* and *B* of shape (10,000, 128) were initialized. A vector of protein means *m* of length 10,000 was generated by sampling from a Gaussian distribution centered at 12 with standard deviation 1.0. An equally sized vector of protein standard deviations was generated, also by sampling from a Gaussian centered at 1.0 with standard deviation 1.0. Each row (*i*) of *A* was then filled by sampling for a Gaussian distribution with mean *m*_*i*_ and standard deviation *s*_*i*_.

To introduce differential abundance, we increased the means of 10% of randomly selected indices such that: *m*_*i*_ = *m*_*i*_ + *m*_*i*_ × *p*, where *p* is a fixed percentage of the mean. This procedure was then repeated by subtracting *m*_*i*_ × *p* for a mutually exclusive 10% of indices. These new *m*_*i*_s were then used to generate quantifications for matrix *B*, again by sampling from a Gaussian centered at each *m*_*i*_

Missingness was then introduced with the MNAR procedure described in the “Dataset Partitioning and Batch Selection” section, in order to achieve missingness proportions of 10%, 20%, 40%, and 60%. For the 40% and 60% conditions, additional MCAR missingness was introduced to achieve the desired proportion.

### Protein Complex Analysis

We compared within-complex to noncomplex protein–protein correlations. The first step was to convert ENSEMBL v44 protein IDs to the Hugo Gene Nomenclature Committee IDs within our joint quantifications matrix. We then divided our Lupine-imputed joint quantifications matrix into 10 subsets according to the CPTAC cohort. Proteins with initial (preimputation) missingness fractions *>*0.5 were removed from these matrices. Each cohort matrix was then subset to only tumor samples. We then searched Hugo Gene Nomenclature Committee IDs against the CORUM database (https://mips.helmholtz-muenchen.de/corum/#download, file name: humanComplexes.txt) of known human protein complexes (39) for each cohort. For each CORUM-annotated complex, we computed the Spearman's correlations between every pair of subunits in the complex. We then computed the same number of Spearman's correlations for pairs of randomly selected proteins from the cohort matrix.

## Results

### Overview of the Lupine Model

The Lupine model takes as input a partially completed protein-by-sample matrix of quantifications and trains a DNN to complete the matrix by filling in missing values. Lupine has two primary components. First, the model uses two linear embedding layers to learn low-dimensional representations of proteins and MS samples, referred to as protein and sample factors, respectively (Fig. 1*A*). Second, for each missing value, the corresponding protein and sample factors are concatenated and fed into a multilayer perceptron, which generates a protein intensity prediction. The MSE between model predictions and training set observations is calculated, and the model weights and biases are updated *via* backpropagation. This process repeats until the model converges. Given the observation that ensembles of individual models, trained with different hyperparameters, often outperform single models (40), Lupine is an ensemble of a user-specified number of individual models.

### Lupine Outperforms the Current State of the Art for TMT Imputation

We hypothesized that, when trained on a large protein-by-samples matrix derived from many different experiments, Lupine would outperform state-of-the-art methods for MS imputation. Accordingly, we constructed a “joint” quantifications matrix from 10 CPTAC datasets (*i.e.*, cohorts) corresponding to 10 types of cancer. Rows in the matrix were proteins, and columns were demultiplexed TMT samples. We partitioned this matrix into train and test sets with an MNAR procedure described in the study by Harris *et al*. (20) and the “Dataset Partitioning and Batch Selection” section. This resulted in a test set that was left skewed relative to the training set (Fig. 1*B*). During model training, we used a “biased” batch selection procedure that preferentially selected matrix entries from the low end of the distribution of intensities for the training set (supplemental Fig. S1). We reasoned that the model would train more effectively if the training set better resembled the test set.

For comparison, we benchmarked Lupine against DreamAI and Gaussian random sampling imputation. DreamAI is the current state of the art for TMT imputation (22) whereas Gaussian random sampling is the most commonly used method (20). We also included MissForest and kNN, two widely used and high-performing methods (20). MNAR missingness was simulated, and missing values were predicted with each method.

**Fig. 2:**
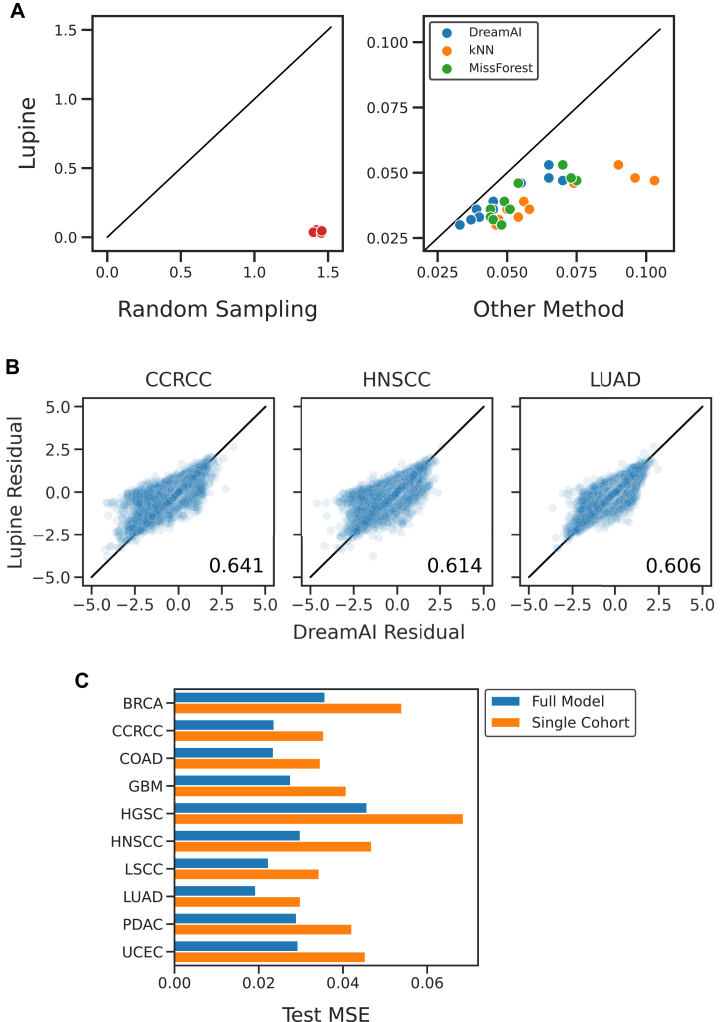
**Benchmarking Lupine against state of the art and commonly used proteomics imputation methods.***A,* test set MSE for Lupine *versus* Gaussian random sampling *(left*) and DreamAI, MissForest, and kNN imputation (*right*), for 10 CPTAC cohorts. *B,* scatterplots of the residuals between model predictions and ground truth (*i.e.*, test set) protein quantifications for Lupine and DreamAI, for three cohorts. The fraction of proteins for which Lupine predictions are more accurate than DreamAI is indicated. *C,* test set MSE for Lupine models trained on the full joint quantifications matrix *versus* individual CPTAC cohorts. CPTAC, Clinical Proteomics Tumor Atlas Consortium; kNN, *k*-nearest neighbor; MSE, mean squared error.

This benchmarking experiment suggests that Lupine indeed outperforms the four baseline methods. For all 10 CPTAC cohorts, the test set reconstruction MSE was lower for Lupine than for any competing method (Fig. 2*A*). The residuals between imputed and observed test set values are shown for Lupine and DreamAI in Figure 2*B*, for three representative cohorts. Points on the diagonal indicate identical predictions made by the two models. Points off the diagonal and centered about 0.0 on the *y*-axis indicate proteins that were more accurately imputed by Lupine. We calculated the fraction of proteins that are more accurately predicted by Lupine than DreamAI. These fractions were 0.641 for clear cell renal cell carcinoma, 0.614 for head and neck squamous cell carcinoma, and 0.606 for lung adenocarcinoma. So as not to bias this analysis by proteins with very low prediction errors, this analysis was limited to proteins, for which either method’s residual was *>*0.25. We conducted a version of this experiment with an MCAR partition instead of MNAR (supplemental Fig. S3). In the MCAR context, Lupine’s performance is comparable to DreamAI, slightly better than MissForest, and considerably better than kNN and Gaussian random sampling. This slight drop in relative performance is not surprising, given Lupine’s batch selection procedure is explicitly designed to accommodate MNAR.

Because individual Lupine models are relatively large, containing 4 to 11 million parameters, we hypothesized that Lupine would perform best when applied to larger datasets. We therefore compared the model’s performance when applied to each individual cohort *versus* the full joint quantifications matrix, using a fixed test set for each comparison. As expected, we observed that Lupine performs better when trained on the full joint quantifications matrix (Fig. 2*C*, *p* = 0.0035, paired *t* test). On average, the MSE decreases by 34% when comparing the single cohort to joint quantifications matrix performance. This result indicates that including additional MS samples in the training set improves performance even on the original test set samples. This result likely reflects the DL principle that more training data are generally better (40)—the full model may have less of a tendency to overfit.

We performed an ablation experiment to assess the effects of Lupine’s ensembling. Models were fit with different random seeds and different numbers of protein factors, sample factors, hidden layers, and nodes per hidden layer. We observe a 40% performance gain from ensembling 10 models compared with a single model (supplemental Fig. S2). The performance gain from ensembling 40 models relative to 10 is only 6%. This finding informed our decision to set the default number of models to 10 in the Lupine Python package.

It is worth bearing in mind that DreamAI is also an ensembling strategy that averages predictions from six individual imputation methods. DreamAI uses a bootstrap aggregation (*i.e.*, bagging) strategy that repeatedly samples the training data, fits models to each bootstrapped set, and averages across sets. Thus, it is reasonable to compare a Lupine ensemble to DreamAI. We attempted to fit DreamAI to the full joint quantifications matrix to include as a baseline in supplemental Fig. S2. However, this proved computationally intractable, because fitting DreamAI to this very large matrix is extremely time consuming.

Lupine also outperforms the four baseline methods on DIA and LFQ datasets (Table 2). Both these datasets are very large, and Lupine was fit to them individually, excluding the joint quantifications matrix construction step. The test set reconstruction error is lowest for Lupine, indicating that for very large datasets, a simple Lupine ensemble model may be sufficient to outperform non-DL competitors.

**Table 2:** Benchmarking Lupine’s performance on non-TMT datasets

Dataset	Type	Lupine	DreamAI	kNN	MissForest	Random sampling
ProCan	DIA	0.35	0.42	0.55	0.45	3.23
MoA	DDA-LFQ	0.76	0.83	0.99	0.81	10.94

DDA, data-dependent acquisition.

Test set mean squared error is indicated. Missing not at random partitions were used to simulate missingness. Twenty percent of observed quantifications were held out for testing.

### Lupine Identifies Additional DA Proteins

The final goal of MS proteomics experiments is often to identify proteins that are DA between experimental groups. This step can be hindered by excessive missingness, which can compromise statistical power, make it impossible to identify low-abundance DA proteins, and lead to the identification of fewer than expected DA proteins. Imputation can help alleviate this problem.

**Fig. 3:**
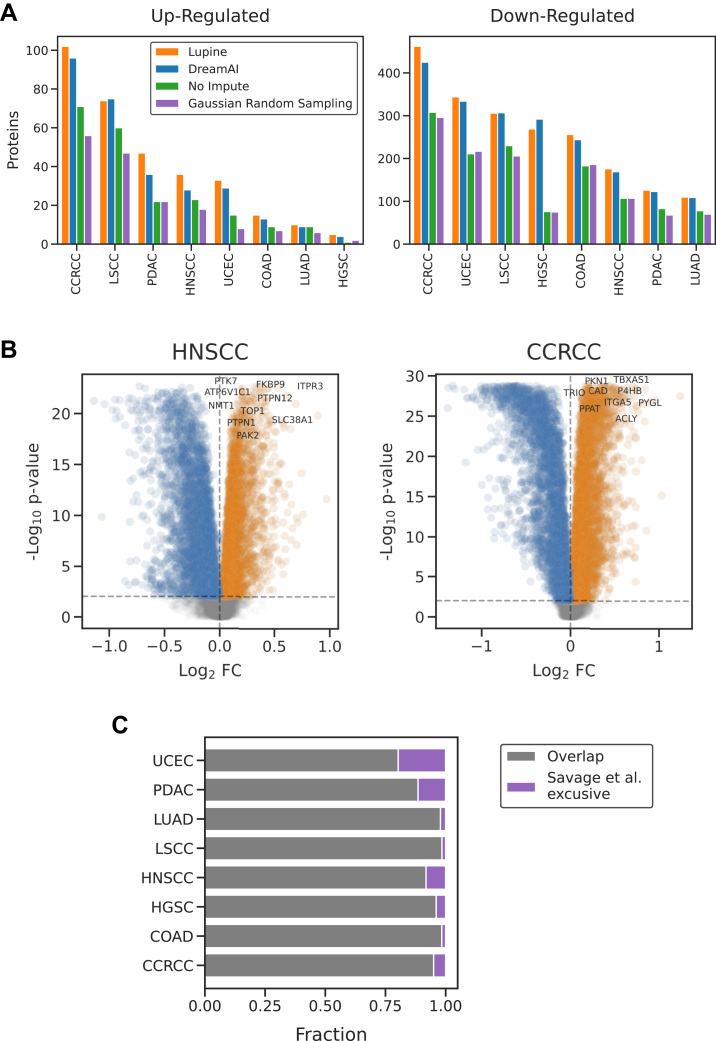
**Identifying differentially abundant (DA) proteins between tumor and nontumor samples.***A,* the number of upregulated and downregulated proteins identified within each CPTAC cohort after imputation with Lupine, DreamAI, Gaussian random sampling, or *no imputation*. *B,* Volcano plots of DA proteins identified after Lupine imputation for two cohorts. Plots have been annotated with the 10 most significantly DA proteins identified by Savage *et al.* (12). *C*, the fraction of overlap between Savage *et al*. and Lupine-imputed DA proteins. CPTAC, Clinical Proteomics Tumor Atlas Consortium.

We compared DA proteins identified between tumor and nontumor samples after imputation with several methods. For seven of eight cohorts, Lupine identifies the most upregulated proteins, and for six of eight cohorts, Lupine identifies the most downregulated proteins (Fig. 3*A*). Two cohorts, breast cancer and glioblastoma, were excluded because of quality control issues with the matched nontumor samples. These cohorts were also excluded from DA analysis in the original CPTAC publications (12, 41).

The DA proteins identified after imputation with Lupine show good concordance with a recent study conducted by CPTAC (Fig. 3*C*). Savage *et al*. (12) used unimputed TMT data to identify potential drug targets. Figure 3*C* plots the percentage overlap between DA proteins annotated by Savage *et al*. and our study. For six of eight cohorts, the overlap is greater than 90%. This experiment serves as a sanity check, assuring us that expected DA proteins are still identified after imputation with Lupine. Note that the DA protein sets reported by Savage *et al*. fulfill two criteria: DA between tumor and nontumor samples and critical for tumor survival and proliferation as determined by a CRISPR knockout screen in cancer cell lines. Figure 3*C* does not include the set of DA proteins identified by only Lupine because of the possibility that these proteins were also identified as DA by Savage *et al*. but were not determined essential by their CRISPR screen. The top-10 most DA proteins identified by Savage *et al*. are also highly DA in our analysis (Fig. 3*B*).

Lupine uniquely identifies DA proteins that may be of biological interest. Most of these proteins are relatively of low intensity (supplemental Fig. S4). These include the protease inhibitor SERPINB7 for lung adenocarcinoma, angiogenesis-associated growth factor vascular endothelial growth factor-C for pancreatic ductal adenocarcinoma, key developmental transcription factor SOX4 for lung squamous cell carcinoma, homeobox protein HOXB4 for uterine corpus endometrial carcinoma, cell–cell adhesion glycoprotein CDH4 for clear cell renal cell carcinoma, and Ras oncogene family member RAB40C in head and neck squamous cell carcinoma. In addition, we conducted gene set enrichment analysis both with and without Lupine imputation. Table 3 provides GO terms that are only significantly upregulated after Lupine imputation. These include cancer-associated GO terms related to DNA replication, immune system recruitment, cell–cell signaling, and blood vessel morphogenesis. It is possible that Lupine enables quantification of low-abundance proteins in cancer-associated genes, increasing statistical power and nudging these GO terms into significance.

**Table 3:** Enriched GO terms among upregulated proteins for tumor *versus* nontumor samples

Cancer type	Enriched gene sets
BRCA	Positive regulation of nucleobase-containing compound metabolic process (GO: 0045935), positive regulation of macromolecule biosynthetic process (GO: 0010557)
CCRCC	Positive regulation of lymphocyte activation (GO: 0051251), cellular response to cytokine stimulus (GO: 0071345)
COAD	Autocrine signaling (GO: 0035425), neutrophil migration (GO: 1990266)
GBM	Cellular response to tumor necrosis factor (GO: 0071356), cellular defense response (GO: 0006968), blood vessel morphogenesis (GO: 0048514), and positive regulation of cell differentiation (GO: 0045597)
LSCC	Mitotic DNA replication (GO: 1902969), DNA damage response (GO: 0006974), protein localization to condensed chromosome (GO: 1903083)
LUAD	Regulation of osteoblast proliferation (GO: 0033688)
PDAC	Response to external stimulus (GO: 0009605)

Abbreviations: BRCA, breast cancer; CCRCC, clear cell renal cell carcinoma; COAD, colon adenocarcinoma; GBM, glioblastoma; LSCC, lung squamous cell carcinoma; LUAD, lung adenocarcinoma; PDAC; pancreatic ductal adenocarcinoma.

The GO terms listed are only significantly enriched (*q* value *<*0.05) after imputing with Lupine. Three CPTAC cohorts did not reveal any unique GO terms after Lupine imputation.

We stress that the DA proteins and GO terms identified after Lupine imputation should be considered in the context of hypothesis generation. For investigative experiments that are tolerant of false positives—for example, identifying potential drug targets in tumor samples—Lupine’s ability to identify DA proteins may prove invaluable. But the gold standard for DA analysis remains directly observed (*i.e.*, nonimputed) quantifications from targeted assays.

**Fig. 4:**
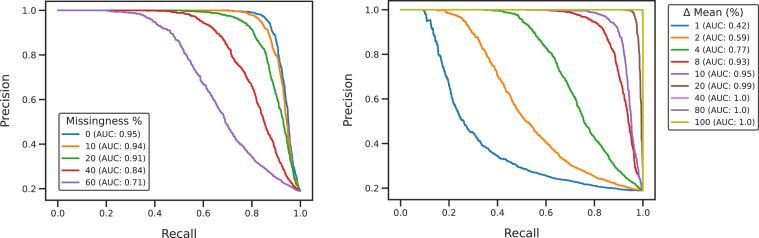
**Lupine identifies differentially abundant (DA) proteins in simulated protein quantification data.** Two experimental groups were simulated with 20% DA proteins between them. *Left*, the missingness fraction was varied. *Right*, the difference in mean (Δ) between the two groups was varied, for only the DA proteins. Missingness was fixed at 20%. The Δ mean for the left plot was fixed at 7.5%.

We evaluated Lupine’s ability to identify DA proteins in simulated protein quantifications data (Fig. 4). We conducted two simulation experiments. In the first, we simulated two conditions with 128 MS runs each, and randomly selected 20% of proteins to be DA between conditions. For these DA proteins, we varied the means between conditions by 7.5%. We then simulated primarily MNAR missingness ranging from 0% to 60%. We calculated the precision-recall area under the curve (AUC) to assess Lupine’s ability to correctly identify DA proteins given various missingness fractions. The AUC for 0% missingness is 0.95, 10% is 0.94, and 20% is 0.91 (Fig. 4*A*). For 60%, the AUC decreased to 0.71.

The second simulation experiment resembled the first except that the missingness fraction was fixed at 20%, and the difference between means (Δ) was varied. When the Δ mean is greater than or equal to 40%, Lupine has a precision-recall AUC of 1.0. The AUC decreases to 0.95 for 10% and 0.77 for 4% (Fig. 4*B*). A Δ mean of 4% represents an extreme case where there is virtually no difference in protein abundance between the two conditions. Any imputation method—or data processing workflow more generally—would struggle to identify DA proteins in this context. Overall, this result gives us confidence that Lupine can identify real and expected DA proteins between groups of samples.

### Within-Complex Correlations Are Higher than Noncomplex Correlations

Many proteins function within larger protein complexes and should therefore have correlated abundances. In principle, a poor imputation method could introduce noise and degrade these correlations. We tested whether within-complex correlations are higher than noncomplex correlations after imputation with Lupine. We used protein complex annotations from the CORUM (39) database. For each cohort, we calculated the Spearman's correlations of intensities for all pairs of proteins within the same CORUM complex, and for the same number of pairs of randomly selected proteins. This analysis was undertaken with and without Lupine imputation. The same number of pairs of proteins was compared before and after Lupine imputation.

**Fig. 5:**
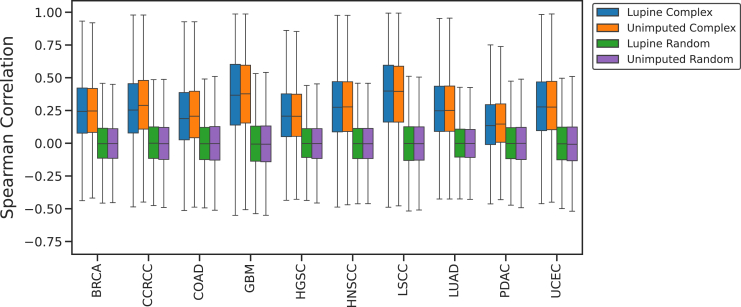
**Comparing within-complex to randomly selected protein–protein correlations before and after Lupine imputation.** Protein–protein intensity correlations for proteins putatively in the same complex (*blue* and *orange*) *versus* randomly selected (*green* and *purple*). Analysis was performed first without imputation, then with Lupine imputation.

Within-complex correlations are significantly higher than noncomplex correlations both before and after Lupine imputation (Fig. 5, paired *t* test *p* values *<*0.01). For Lupine-imputed data, the mean within-complex correlation is 0.27, compared with 0.00 for noncomplex. For unimputed data, the within-complex correlation is 0.28, compared with 0.00 for noncomplex. It is reassuring that Lupine does not reduce within-complex correlations and that noncomplex correlations remain virtually zero after imputation. This result suggests that Lupine is not learning spurious correlations between proteins.

### Lupine Learns a Representation of Proteins and MS Samples

**Fig. 6:**
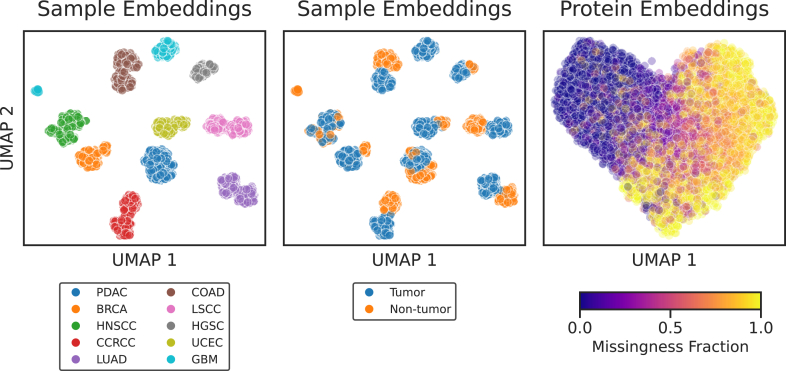
**UMAP projections of Lupine’s learned embedding space.***Left,* points correspond to patient samples and are colored by CPTAC cohort. *Center,* points correspond to patient samples and are colored by sample type. *Right,* points correspond to proteins and are colored by protein missingness fraction in the joint quantifications matrix. CPTAC, Clinical Proteomics Tumor Atlas Consortium; UMAP, uniform manifold approximation and projection.

Lupine, like many DL models, learns in part by projecting observed inputs into a latent embedding space. We hypothesized that if Lupine is learning effectively, then this embedding space should correspond to some aspects of the biological signal rather than noise. We examined the latent embeddings of a Lupine model fit to the CPTAC joint quantifications matrix by reducing the embedding dimensionality with uniform manifold approximation and projection and then coloring the 2D projection using various classes of metadata. We found that Lupine’s sample embeddings cluster exclusively by CPTAC cohort, with separate clusters for tumor and nontumor samples (Fig. 6). Again, Lupine does not have access to metadata, meaning these relationships are learned directly from the protein quantifications. The protein embeddings do not form separate clusters but do exhibit a clear gradation by protein missingness fraction. We tried coloring the protein embeddings by physicochemical properties, including size, charge, and hydrophobicity, but this analysis did not reveal any clear trends. Future work will continue to explore this learning embedding space. It may be interesting to color by cancer subtypes or to search for outlier patient samples that cluster disconcordantly with their clinical annotations.

## Discussion

Lupine is a proteomics imputation method that learns from multiple datasets at once. Lupine was trained on a joint protein quantifications matrix consisting of 10 separate datasets for a total of 18,162 proteins across 1755 MS samples. Our experiments demonstrate that Lupine performs better when trained on this joint quantifications matrix than on any of the 10 individual datasets (Fig. 2*C*). We speculate that a Lupine model trained on an even larger training set will perform even better. Lupine is distinct among imputation methods in that it is designed to learn from many experiments; existing methods consider a single MS experiment at a time.

We also see evidence that Lupine, while originally developed for imputation of large-scale TMT datasets, may work equally well on other proteomics modalities. We benchmarked Lupine on several very large DIA and LFQ datasets and observed state-of-the-art performance. Future work will examine whether a Lupine-style model applied to a heterogeneous training set consisting of MS runs from many different sample types and acquisition strategies may perform even better. We speculate that, given its ability to coembed MS runs from different sample types (Fig. 6), Lupine can also learn to coembed different MS acquisition strategies, while still capturing biological differences between samples. While our analysis was primarily focused on CPTAC data, Lupine is not limited to cancer proteomics. Lupine may be used to impute peptide or protein quantifications from any acquisition strategy or sample type.

Lupine is a Python package available on GitHub (https://github.com/Noble-Lab/lupine) with an MIT open-source license. The Python package includes a command that allows users to attach their own MS samples to our existing joint quantifications matrix. Lupine may then be fit to the modified joint quantifications matrix to impute the user’s samples. Given the relationship between performance and number of ensembled models (supplemental Fig. S2), the Python package defaults to an ensemble of 10. The Lupine-imputed CPTAC protein quantification matrices produced by this study are available on Zenodo (https://zenodo.org/records/13146445). We hope the community will continue to mine these imputed MS samples for insights into cancer biology.

Finally, future work will focus on attaching statistical measures of confidence to imputed values. Because these protein quantitations are not actually measured by the instrument, they should be thought of as lower quality than observed quantitations. However, most researchers do not take this into account when performing downstream analysis and effectively treat imputed and observed quantitations the same. Prediction-powered inference is a statistical framework for attaching confidence intervals to DL or machine learning predictions (42). A powerful extension of this work would be to build a prediction-powered inference framework that allows researchers to prioritize the higher-confidence observed values in downstream analysis, while still benefiting from the increased statistical power and low-abundance quantifications afforded by Lupine’s imputation.

## Data Availability

Unimputed CPTAC datasets were obtained from the Proteomics Data Commons web portal (https://pdc.cancer.gov/pdc/cptac-pancancer). The name of the file we accessed was Proteome UMich GENCODE34 v1.zip. The Lupine-imputed versions of these protein quantifications are available at https://zenodo.org/records/13146445.

The code used to generate all the figures in this article can be found at https://github.com/Noble-Lab/2024_proteomics_impute. The Python package implementing Lupine can be found at https://github.com/Noble-Lab/lupine.

## Supplemental data

This article contains supplemental data.

## Conflict of interest

The authors declare no competing interests.
