# Supplementary material for: Proteomics Data Imputation With a Deep Model That Learns From Many Datasets
Source: Mol Cell Proteomics. 2025 Nov 18;24(12):101461. doi: 10.1016/j.mcpro.2025.101461 (PMC12753234; doi:10.1016/j.mcpro.2025.101461)
Supplement: Supplemental data [file mmc1.pdf]

Supplement to "Proteomics data imputation with a deep model that learns from many datasets"

Lincoln Harris[1] and William S. Noble[1,2]

[1]Department of Genome Sciences, University of Washington
[2]Paul G. Allen School of Computer Science and Engineering, University of Washington

September 22, 2025

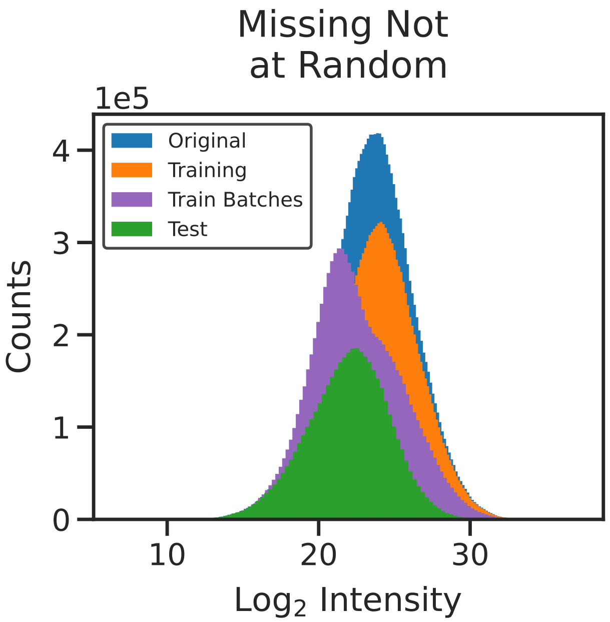

Supplementary Figure 1. **Lupine used a biased batch selection procedure during model training.** The joint quantifications matrix (blue) was partitioned into training (orange) and test (green) sets with an MNAR procedure described in Section 2.2. Training batches were preferentially selected from the low-end of the training set distribution; repeated sampling of the training set was allowed.

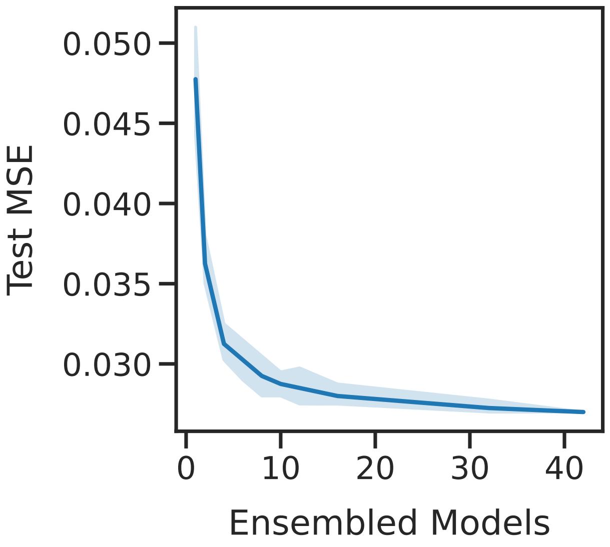

Supplementary Figure 2. **Accuracy as a function of the number of ensembled Lupine models.** Lupine models were fit to the training set derived from the joint quantifications matrix. Hyperparameters were selected as described in Section 2.4. Four independent sets of ensembled models were fit to obtain the 95% confidence intervals shown.

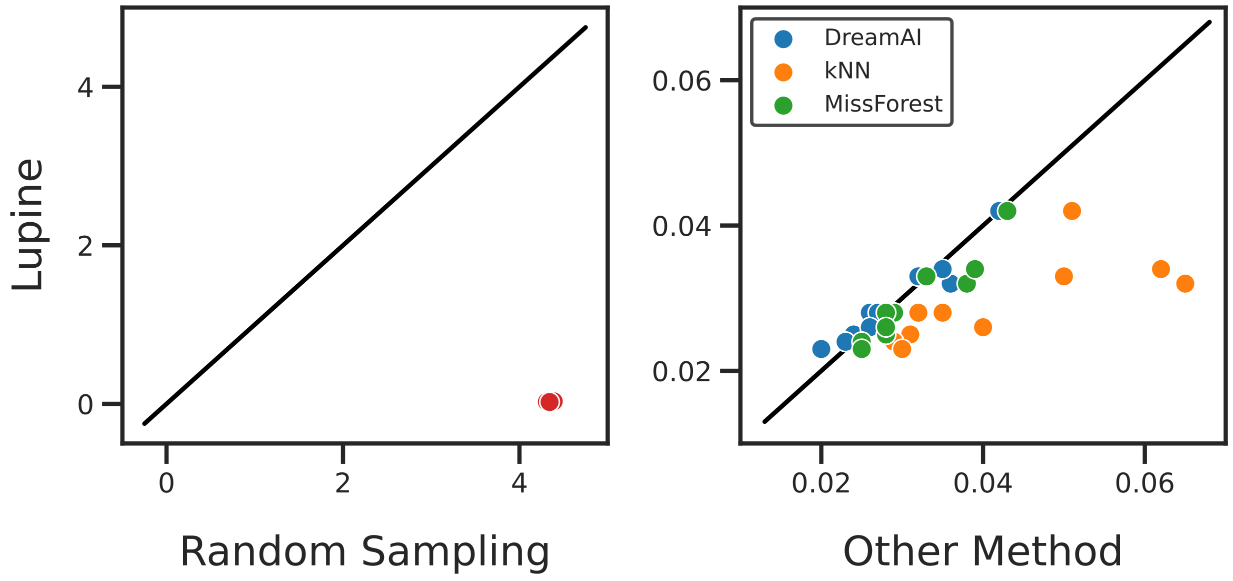

Supplementary Figure 3. **Benchmarking imputation methods on CPTAC data after an MCAR partition.** 20% of present observations were withheld for testing. Each dot represents a CPTAC cohort. For practical reasons, MissForest was limited to 4 training epochs. kNN was trained with $k = 8$ neighbors.

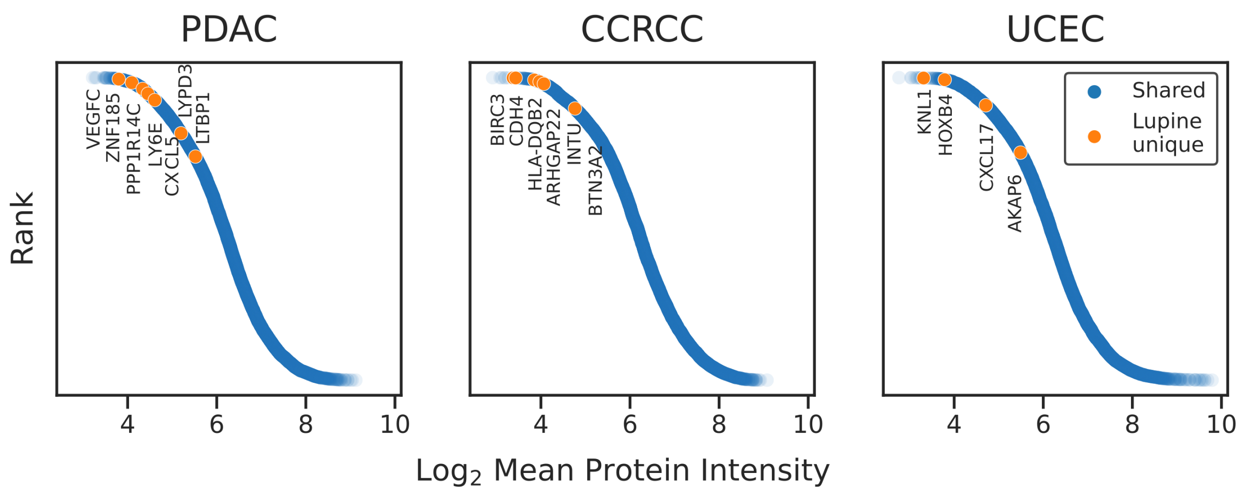

Supplementary Figure 4. **Rank plots of mean intensities of DA proteins identified between tumor and non-tumor samples.** Blue indicates DA proteins identified after imputation with at least two of Lupine, DreamAI and Gaussian random sampling. Orange indicates DA proteins uniquely identified after Lupine imputation. The mean protein intensities for non-tumor samples are shown. Proteins with >90% initial missingness were excluded from this analysis.

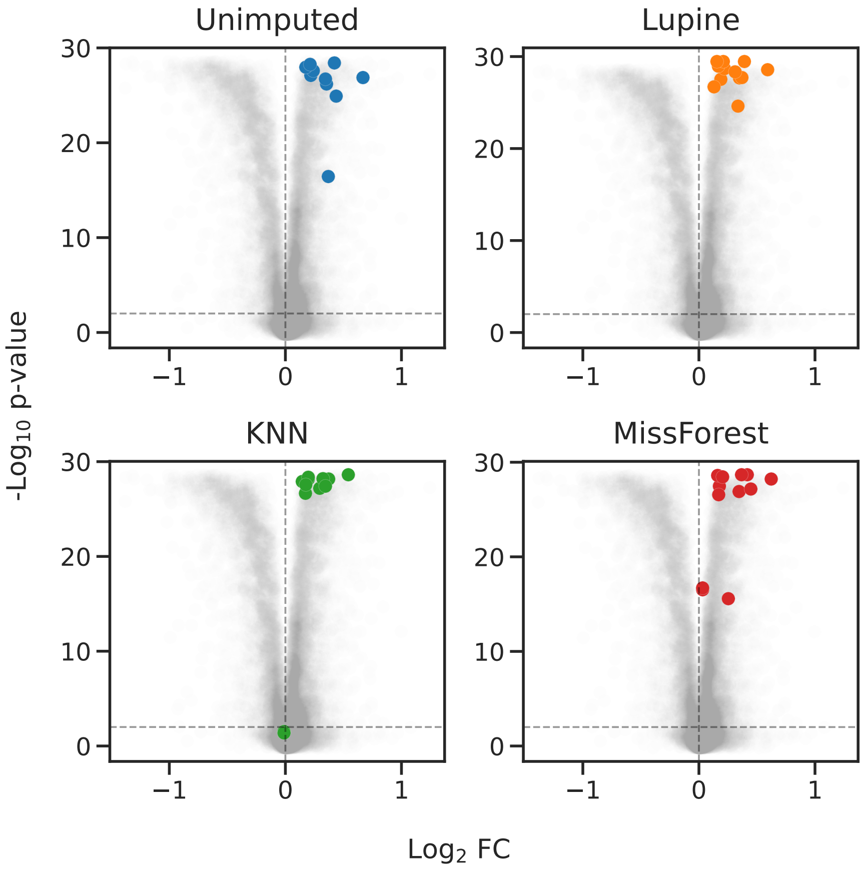

Supplementary Figure 5. **Lupine recovers the top-10 DA proteins after introducing additional missingness.** 40% additional missingness was introduced into only the top-10 DA proteins. Top-10 DA proteins were previously determined by Savage et al. These top-10 proteins are indicated on each volcano plot. Imputation was performed with Lupine, kNN and MissForest. For the *unimputed* comparison, the 40% missingness simulation was not performed.

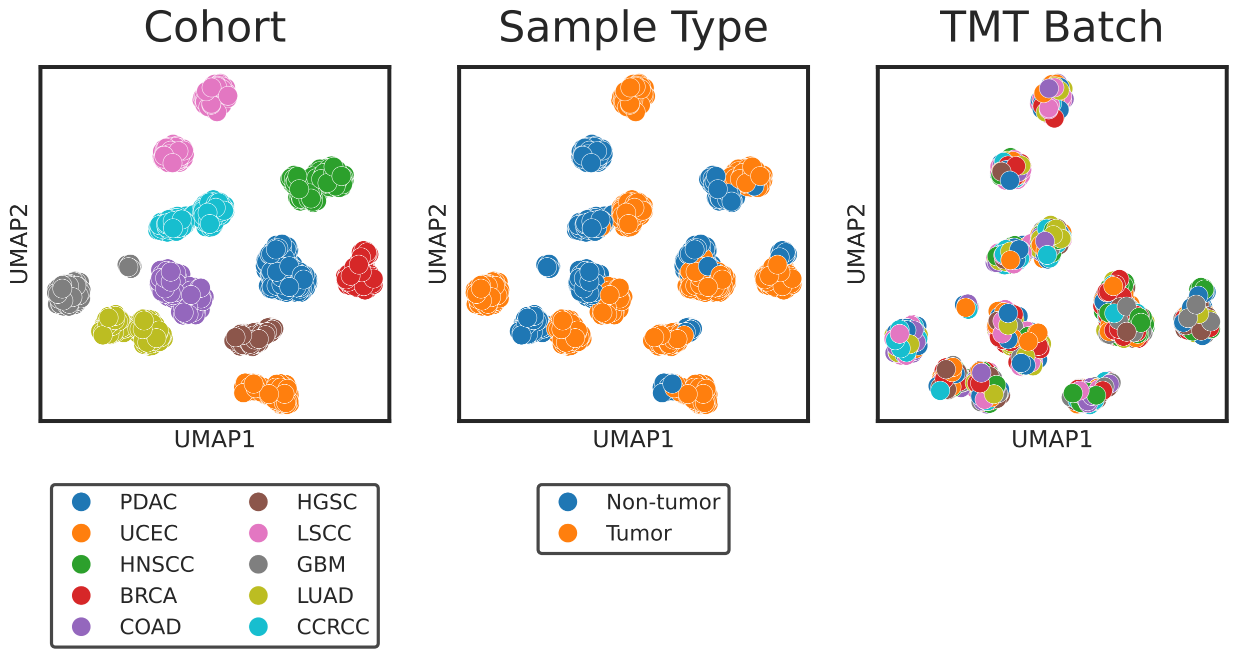

Supplementary Figure 6. **UMAP projections of the full joint quantifications matrix, following Lupine imputation.** Left: colored by CPTAC cohort; center: colored by sample type; right: colored by TMT batch ID. Each point corresponds to an MS sample.
